# Cancer cell immunity-related protein co-expression networks are associated with early-stage solid-predominant lung adenocarcinoma

**DOI:** 10.3389/fonc.2024.1273780

**Published:** 2024-02-21

**Authors:** Toshihide Nishimura, Ákos Végvári, Haruhiko Nakamura, Kiyonaga Fujii, Hiroki Sakai, Saeko Naruki, Naoki Furuya, Hisashi Saji

**Affiliations:** ^1^ Department of Chest Surgery, St. Marianna University School of Medicine, Kawasaki, Japan; ^2^ Division of Chemistry I, Department of Medical Biochemistry and Biophysics, Karolinska Institutet, Biomedicum, Stockholm, Sweden; ^3^ Laboratory of Analytical Chemistry, Daiichi University of Pharmacy, Fukuoka, Japan; ^4^ Department of Pathology, St. Marianna University Hospital, Kawasaki, Japan; ^5^ Division of Respiratory Medicine, Department of Internal Medicine, St. Marianna University School of Medicine, Kawasaki, Japan

**Keywords:** solid predominant adenocarcinomas of the lung, WGCNA, data-driven co-expression protein networks, upstream regulator analysis, laser microdissection, proteomics, mass spectrometry

## Abstract

**Background:**

Solid-predominant lung adenocarcinoma (SPA), which is one of the high-risk subtypes with poor prognosis and unsatisfactory response to chemotherapy and targeted therapy in lung adenocarcinoma, remains molecular profile unclarified. Weighted correlation network analysis (WGCNA) was used for data mining, especially for studying biological networks based on pairwise correlations between variables. This study aimed to identify disease-related protein co-expression networks associated with early-stage SPA.

**Methods:**

We assessed cancerous cells laser-microdissected from formalin-fixed paraffin-embedded (FFPE) tissues of a SPA group (*n* = 5), referencing a low-risk subtype, a lepidic predominant subtype group (LPA) (*n* = 4), and another high-risk subtype, micropapillary predominant subtype (MPA) group (*n* = 3) and performed mass spectrometry-based proteomic analysis. Disease-related co-expression networks associated with the SPA subtype were identified by WGCNA and their upstream regulators and causal networks were predicted by Ingenuity Pathway Analysis.

**Results:**

Among the forty WGCNA network modules identified, two network modules were found to be associated significantly with the SPA subtype. Canonical enriched pathways were highly associated with cellular growth, proliferation, and immune response. Upregulated HLA class I molecules HLA-G and HLA-B implicated high mutation burden and T cell activation in the SPA subtype. Upstream analysis implicated the involvement of highly activated oncogenic regulators, MYC, MLXIPL, MYCN, the redox master regulator NFE2L2, and the highly inhibited LARP1, leading to oncogenic IRES-dependent translation, and also regulators of the adaptive immune response, including highly activated IFNG, TCRD, CD3-TCR, CD8A, CD8B, CD3, CD80/CD86, and highly inhibited LILRB2. Interestingly, the immune checkpoint molecule HLA-G, which is the counterpart of LILRB2, was highly expressed characteristically in the SPA subtype and might be associated with antitumor immunity.

**Conclusion:**

Our findings provide a disease molecular profile based on protein co-expression networks identified for the high-risk solid predominant adenocarcinoma, which will help develop future therapeutic strategies.

## 1 Introduction

Lung cancer is the leading cause of death globally, of which lung adenocarcinoma is the most common pathological subtype. Invasive nonmucinous adenocarcinoma is primarily categorized into five histopathological subtypes: lepidic, acinar, papillary, micropapillary, and solid, based on the 2021 World Health Organization Classification of Lung Tumors (1). Micropapillary- and solid-predominant lung adenocarcinoma (MPA and SPA, respectively) are high-risk subtypes with high metastatic potential and the worst prognosis. In contrast, low-risk subtypes characterized by well- and moderately-differentiated morphologies, such as lepidic predominant adenocarcinoma (LPA) have a favorable prognosis (2, 3). Lung adenocarcinomas usually contain complex mixtures of different subtypes. MPA exhibits a micropapillary pattern, which is the primary histological pattern assessed semi-quantitatively in 5% increments in resected specimens. It is associated with lymphatic invasion, pleural invasion, and lymph node metastases (4).

Caso et al. conducted a genomic characterization of prognostically important predominant histologic subtypes of lung adenocarcinoma and found that MPA and SPA exhibited a higher tumor mutational burden, increased chromosomal instability (CIN), higher APOBEC (the enzyme with DNA mutagenesis function) mutational signatures, more oncogenic pathway alterations, and the lowest frequency of targetable genomic alterations among the subtypes (5). Micropapillary or solid patterns are risk factors for predicting poor recurrence-free survival in early-stage IA lung adenocarcinoma (6). Recently, Jeon et al. compared the clinicopathological features and clinical course of patients with the MPA and SPA subtypes, including predominant and non-predominant subtypes after curative resection of stage I lung adenocarcinoma and analyzed the prognostic factors. The clinical results were different for stage I high-grade adenocarcinoma and the predominant micropapillary subtype was an independent prognostic factor for recurrence, whereas the solid subtype was a significant factor for overall survival (7).

A pivotal challenge is to unravel the underlying cancer biology of those high-risk lung adenocarcinomas and how tumor morphologic appearances are linked to malignant clinical outcomes. Solid predominant adenocarcinoma (SPA) shows a major component of polygonal tumor cells forming sheets that lack recognizable patterns of adenocarcinoma, such as acinar, papillary, micropapillary, or lepidic growth. SPA subtype and the presence of solid pattern are associated with numerous poor prognostic factors, including higher mitotic count, high risk of occult lymph node metastases, thyroid transcription factor-1 (TTF-1) negativity, and less frequent epidermal growth factor receptor (EGFR) mutations (8). However, molecular profiles characterizing the SPA subtype remain unclear. In this study, we focus on identifying disease-related co-expression protein networks and their upstream regulators associated with the SPA subtype.

Mass spectrometry (MS)-based proteomics has proven feasible in the identification and quantification of proteins expressed in clinical specimens. Quantitative proteome data can be used to identify key disease-related proteins and therapeutic targets (9). We have adopted label-free spectral counting-based semiquantitative MS-based proteomics, following the collection of target cancerous cells from formalin-fixed paraffin-embedded (FFPE) tumor specimens by laser microdissection (LMD). This study aimed at identifying the co-expression protein networks associated with the early-stage high-risk lung adenocarcinoma SPA, by comparing the early-stage low-risk subtype LPA and another high-risk subtype MPA. Upstream regulator and causal network analysis (10) were performed for data-driven protein co-expression networks significant to SPA, obtained by the weighted gene co-expression network analysis (WGCNA), an unsupervised clustering method based on the correlation network expression (11), applied to quantitative proteome datasets.

## 2 Materials and methods

### 2.1 FFPE tissue specimens and sample preparation

**Figure 1:**
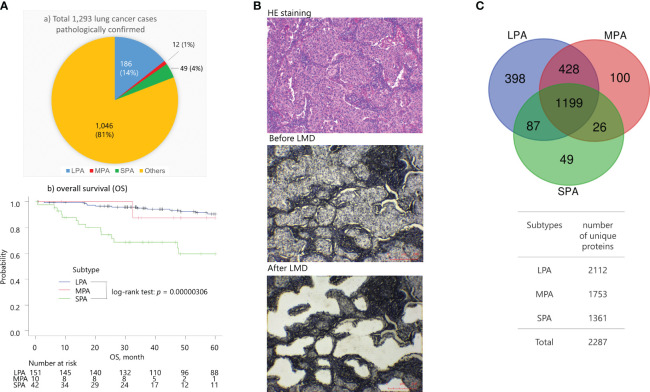
**(A)** 1,293 lung cancer patients were pathologically confirmed and received surgical operations between January 2008 to April 2022. at the St. Marianna University School of Medicine Hospital (Kawasaki, Kanagawa, Japan) The proportions of subtypes were LPA (14%), MPA (1%), SPA (4%), and others (81%). The SPA cases showed a worse overall survival (OS) compared with the LPA cases (log-rank test: *p* = 3.06 × 10^-5^), whereas the MPA cases were not statistically significant due to the limited number of cases against the LPA and SPA cases. **(B)** Representative images of tumor tissues from solid predominant adenocarcinomas (SPA) (sample SPA_T21) stained with hematoxylin & eosin (HE) using standard histological methods, and only with hematoxylin prior to and after laser microdissection (LMD). Scale bars in red color are indicated at the bottom right. **(C)** Venn map of the identified proteins.

Of 1,293 patients who underwent surgery for lung cancer at St. Marianna University Hospital between 2000 and 2020, 186 (14%), 12 (1%), and 49 (4%) had tumors that were histologically confirmed to represent the LPA, MPA, and SPA subtypes, respectively (**Figure 1A**). SPA, a high-risk subtype, was contrasted to LPA, a low-risk subtype. The pathological specimens were independently reviewed by two pathologists (H. N. and S. N.) to confirm that they fulfilled the 2015 World Health Organization classification criteria for lung tumors (histological criteria) (12). FFPE tumor tissue blocks from 12 surgical specimens histologically confirmed as lung LPA, MPA, and SPA were obtained without patient identifiers from the St. Marianna University School of Medicine Hospital. Informed consent was obtained from all participating subjects. The protocol was approved by the Institutional Review Board of St. Marianna University School of Medicine (approval no. 1461) and the study adhered to the Helsinki Declaration.

For tissue microdissection, 10-μm-thick sections from the FFPE tumor blocks were cut and placed on DIRECTOR slides (OncoPlex Diagnostics Inc., Rockville, MD, USA). The sections were deparaffinized and stained with hematoxylin using standard protocols before dissection. Microdissection was performed using a Leica LMD7 microdissection microscope (Leica, Wetzlar, Germany) (**Figure 1B**). A total area of 4 mm^2^ with approximately 15,000 tumor cells was directly transferred from the FFPE sections via laser dissection into the cap of a 200 μL low-binding tube. Proteins were extracted and digested with trypsin using the Liquid Tissue™ MS Protein Prep kit (OncoPlex Diagnostics Inc.) according to the manufacturer’s instructions (13). Briefly, dried microdissected pellets were suspended in 20 μL of Liquid Tissue buffer and heated at 95°C for 90 min and then cooled on ice for 3 min before the addition of 0.1 μg of trypsin. The tubes were then incubated at 37°C overnight. Dithiothreitol was added to a final concentration of 10 mM and the samples were heated for 5 min at 95°C. The digested samples were dried, resuspended in 50 μL of a 2% acetonitrile aqueous solution containing 0.1% trifluoroacetic acid, and stored at −20°C until analysis.

### 2.2 Proteomic analysis by LC-MS/MS

A label-free quantitation approach using spectral counting by LC-MS/MS was adopted for global proteomic analysis. The digested samples (5 μL for a single run) were analyzed in triplicate by LC-MS/MS using a reverse-phase LC system interfaced with a Q Exactive Orbitrap mass spectrometer (Thermo Fisher Scientific, Bremen, Germany) via a nano-electrospray ionization device (AMR Inc., Tokyo, Japan). The mass spectrometer was operated in data-dependent acquisition mode. Full-scan MS spectra were acquired in the range of *m/z* 350–1600 at a resolution of 70,000. The top ten most intense peaks from the survey scan were selected for fragmentation with higher-energy collisional dissociation with a normalized collision energy of 27% and isolation window of *m/z* 1.6. The dynamic exclusion time for precursor ions selected for MS/MS fragmentation was 15 *s*, and the automatic gain control target values for MS and MS/MS were 1 × 10^6^ and 1 × 10^5^, respectively.

The LC system consisted of an Ultimate3000 HPLC System (Thermo Fisher Scientific), a trap cartridge (0.3 mm × 5.0 mm, CERI, Tokyo, Japan), and a capillary separation column (Zaplous column alpha-PepC18, 3 μm, 12 nm, 0.1 mm × 150 mm, AMR Inc.) fitted with an emitter tip (FortisTip, OmniSeparo-TJ, Hyogo, Japan). An auto-sampler (HTC-PAL, CTC Analytics, Zwingen, Switzerland) was used to load the samples into the trap, which was then washed with solvent A (2% acetonitrile aqueous solution containing 0.1% formic acid) to concentrate and desalt the peptides in the trap. Subsequently, the trap was connected in series to the separation column, and the peptides were eluted from the whole column with 0.1% formic acid aqueous solution and acetonitrile using a linear 5%–40% acetonitrile concentration gradient over 90 min at a flow-rate of 500 nL min^−1^.

### 2.3 Protein identification

The raw data were processed using PatternLab for Proteomics software v4.0. Peptide sequence matching (14) was done using the Comet algorithm against the UniProt *Homo sapiens* database. A target-reverse strategy was employed for increased confidence in protein identification. This search considered tryptic peptide candidates as well as the formylation of lysine and oxidation of methionine as variable modifications. The Comet search engine considered a precursor mass tolerance of 40 ppm and a fragment bin tolerance of 0.02 Da. The validity of the peptide spectrum matches was assessed using the Search Engine Processor (SEPro) module of PatternLab. The acceptable FDR for spectra, peptide, and protein were 3%, 2%, and 1%, respectively. The expressions of the identified proteins were assessed using spectral count-based protein quantification. The spectral count represents the number of MS/MS spectra assigned to each protein.

### 2.4 Weighted correlation network analysis

The similarity in protein expression patterns for all protein pairs was calculated according to their pairwise Pearson’s correlation coefficient [i.e., the similarity between proteins i and j was defined as (1-*r*_i,j_)/2, where *r*_i,j_ is the Pearson’s correlation coefficient of the protein expression pattern between the two proteins]. We performed a network topology analysis for various soft-thresholding powers ranging from 1 to 2 to choose an optimal value of balance between independence and mean connectivity. A topological overlap matrix (TOM) that considers topological similarities between a pair of proteins in the network was then generated from the resultant scale-free co-expression network. We generated a tree by hierarchical clustering using dissimilarity according to TOM (1−TOM), and protein modules were determined using dynamic tree-cutting to trim the branches (11).

The modules were summarized by the first principal component, which is referred to as eigen proteins in the text, as they express the highest connectivity in the module. Module membership, defined as the correlation between the protein expression profile and the module eigen-protein, was measured with values ranging from 0 to 1, with “0” representing a gene that is not part of the module and “1” representing high connectivity with the module. Subsequently, the module-trait association was determined using the correlation between the module eigen-protein and the three subtypes: MPA, SPA, and LPA. A protein module was summarized by the top hub protein (referred to as “eigen-protein”) with the highest connectivity in the module. The WGCNA analysis was performed using the WGCNA R-package (11) implemented in RStudio.

### 2.5 Protein-PPI network construction

We used the STRING database (version 11.5) (https://string-db.org/) to construct a PPI network for a protein module (15). STRING networks were calculated under the criteria for linkage with experiments, databases, text mining, and co-expression using the default settings (medium confidence score: 0.400; network depth: 0 interactions). Functional enrichment results were obtained for canonical pathways with a p-value <0.05. Proteins in a module were mapped in the PPI network from the STRING database to produce the results of the enrichment analysis regarding the biological process (GO) and Reactome pathways (HAS). Protein networks were subsequently exported to Cytoscape (version 3.9.1) (Institute for Systems Biology, Seattle, WA, USA: https://cytoscape.org/) (16) from the STRING database. The hub proteins in each module were identified according to their intramodular connectivity and their correlation with module eigen-proteins. The proteins inside the co-expression modules exhibit high connectivity and the proteins within the same module may play similar roles. The top 10 high-degree proteins were identified using the cytoHubba plugin (17). The top-ranked proteins in each module were considered hub proteins and designated “highly connected proteins.” Functional enrichment results were obtained for canonical pathways by considering a network bias-corrected *p*-value of <0.05 for statistical significance.

The multivariate correlation analysis (MVA) of semiquantitative key protein expressions was performed using the JMP software (SAS Institute, Cary, NC, USA), and which result was visualized using the Intervene Shiny App (https://intervene.shinyapps.io/intervene/) (18).

### 2.6 Upstream regulator and causal network analysis by IPA

Upstream regulators, causal networks, and canonical pathways were predicted using IPA software (http://www.ingenuity.com) (10). Quantile-normalized protein expression data of the selected modules were used as input datasets. Both the upstream regulators and causal networks (*p* < 0.05) predicted from the WGCNA network modules were significantly associated with the three subtypes (LPA, MPA, and SPA), in which the activation and the inhibition of a predicted network were defined by *z*-values that were >2.0 and <−2.0, respectively. The upregulation was defined by *z*-values > 1.5 and < 2.0, whereas downregulation was defined by *z*-values > −2.0 and < −1.5.

## 3 Results

### 3.1 Proteome datasets

At the St. Marianna University School of Medicine Hospital (Kawasaki, Kanagawa, Japan), 1,293 lung cancer patients were pathologically confirmed and underwent surgery between January 2008 to April 2022. These cases consisted of the following subtypes: LPA (14%), MPA (1%), SPA (4%), and others (81%) (**Figure 1A**a). The SPA cases exhibited worse overall survival (OS) compared with the LPA cases (log-rank test: *p* = 3.06 × 10^−5^) (**Figure 1A**b), whereas the MPA cases were not statistically significant because of the limited number of samples.

FFPE tissue specimens were obtained by surgical resection of early-stage (IA-IB) lung adenocarcinoma patients who were pathologically confirmed as having solid predominant (SPA, *n* = 5), micropapillary (MPA, *n* = 3), and lepidic (LPA, *n* = 4) subtypes (**Table 1**). Presurgical treatment was not performed for any of the lung adenocarcinoma patients. MS-based proteomic analysis was carried out on approximately 15,000 cancerous cells collected from the FFPE specimens by laser microdissection (LMD) (**Figure 1B**). A total of 2,287 proteins were identified, of which 1,199 (52.4%) were commonly expressed in the cancerous cells. LPA, MPA, and SPA contained 398 (17.4%), 100 (4.4%), and 49 (2.1%) unique proteins, respectively (**Figure 1C**).

**Table 1:** Clinicopathological information of the recruited patients.

Variable	Category	No. patients	%
Gender			
	Female	5	41.7
	Male	7	58.3
Age(y)			
	Average ± SD	64.4 ± 5.5	
Smoking index (Brinkmann Index, BI)			
	Female		
	BI=0	4	80.0
	0 < BI ≤ 400	1	20.0
	400 < BI≤ 600	0	0.0
	600 < BI ≤ 1200	0	0.0
	BI > 1200	0	0.0
	Male		
	BI=0	2	28.6
	0 < BI ≤ 400	1	14.3
	400 < BI ≤ 600	1	14.3
	600 < BI ≤ 1200	1	14.3
	BI > 1200	2	28.6
Histologic type			
	Adenocarcinoma	12	100.0
Subtype			
	Lepidic predominant adenocarcinoma (LPA)	4	33.3
	Micropapillary adenocarcinoma (MPA)	3	25.0
	Solid adenocarcinoma (SPA)	5	41.7
Surgical method			
	Radical lobectomy	11	66.7
	Limited resection	1	33.3
Tumor size on CT			
	T1a (≦1cm)	0	0.0
	T1b (1-2cm)	2	16.7
	T1c (2-3cm)	4	33.3
	T2a (3-4cm)	4	33.3
	T2b (4-5cm)	2	16.7
	T3 (5-7cm)	0	0.0
	T4 (>7cm)	0	0.0
Clinical stage			
	cIA	6	50.0
	cIIA	0	0.0
	cIB	6	50.0
	cIIB	0	0.0
	cIV	0	0.0
*EGFR* mutation status			
	Positive		
	L858R	1	8.3
	Ex19 E746-A750 del	3	25.0
	Negative		
	Neither L858R	8	66.7
	nor Ex19del		

LPA, lepidic predominant adenocarcinoma; MPA, micropapillary adenocarcinoma; SPA, solid adenocarcinoma; BI, Brinkmann Index.

**Figure 2:**
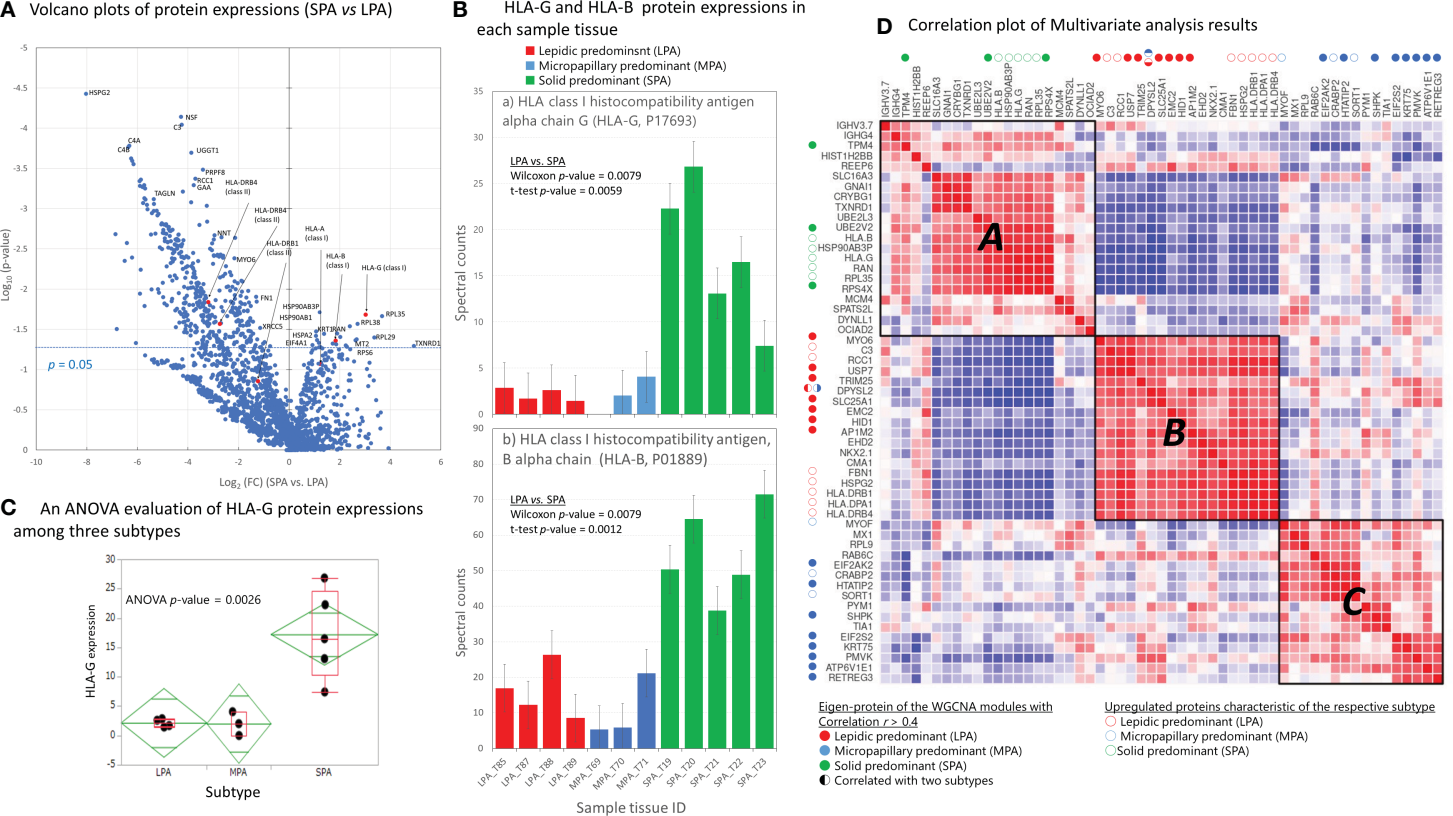
Semi-quantitative protein expression and multivariate analysis of eigen-protein expression. **(A)** Volcano plots of protein expression in SPA *vs*. LPA. **(B)** Expressions of HLA class I proteins, HLA-G and HLA-B, are presented in all the sample tissues. **(C)** An ANOVA test of HLA-G expressions among three subtypes. **(D)** Multivariate correlation analysis (MVA) for the spectral counting-based expression of forty eigen-protein and fifteen proteins characteristic of the respective subtypes, assuming three-group clustering (correlation coefficient: Pearson; heatmap order: hierarchical clustering; agglomeration method: complete; the number of clusters: 3). The eighteen eigen-proteins of the WGCNA modules relatively well correlated (*r* > 0.4) with and upregulated proteins characteristic of respective subtypes are denoted in colored filled-circles and colored circles (LPA: red; MPA: blue; SPA: green).

Overall, 2,112, 1,753, and 1,361 proteins were identified from LPA, MPA, and SPA, respectively. A gene ontology (GO) analysis was performed using the Protein Analysis Through Evolutionary Relationships (PANTHER, version 17.0) program (Paul D. Thomas, University of Southern California, Los Angeles, CA, USA) (19) and revealed similar results among the three subtypes (**Supplementary Figure S1**). Volcano plots were generated from the protein expression data obtained by SimpliFi™ software (PROTIFI, Farmingdale, NY, USA; https://simplifi.protifi.com). Upregulated proteins significant to SPA included large ribosomal subunit protein uL29 (RPL35), human leukocyte antigen G (HLA-G), GTP-binding nuclear protein Ran (RAN), human leukocyte antigen B (HLA-B), and putative heat shock protein HSP90-beta-3 (HSP90AB3P). The expression of HLA-G protein was significantly associated with and highly upregulated in the SPA subtype (**Figure 2A**), which was confirmed by its ANOVA test with a high significance to the SPA subtype with *p*-ANOVA = 0.0026 (**Figure 2C**).

### 3.2 Identification of protein co-expression networks by WGCNA

Weighted correlation network analysis, also known as weighted gene co-expression network analysis (WGCNA) incorporates traditional data exploration techniques, but its intuitive network language and analysis framework go beyond standard analysis techniques. It uses a network approach and is suitable for the integration of complementary genomic/proteomic datasets so that it can be interpreted as a data analysis technique for systems biology (11).

**Figure 3:**
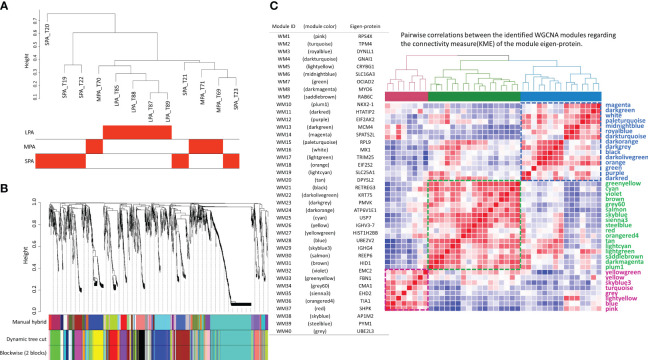
The forty protein network modules identified by weighted gene co-expression network analysis (WGCNA). **(A)** Sample dendrogram and trait heatmap, **(B)** protein cluster dendrogram, and **(C)** pairwise correlations between the 40 identified modules concerning the connectivity measure (KME) of the module eigen-protein (correlation coefficient: Pearson; heatmap order: Euclidean; agglomeration method: complete; the number of clusters: 3), where the color bars represent the three clusters, together with lists of module ID, module color and eigen-protein.

Following hierarchical clustering of the samples based on protein abundance (**Figure 3A**), a WGCNA analysis (11) was performed with a soft threshold power of 10, which was selected to approximate a scale-free topology, a minimum module size of 10, and a module detection sensitivity (*deepSplit*) of 4. Correlations between the resultant modules and the traits were obtained to identify protein modules that were significantly associated with the respective traits. Forty protein modules were identified by clustering all of the proteins and constructing weighted protein co-expression networks. The protein cluster dendrogram is presented in **Figure 3B**. A heatmap of eigen-protein expression for pairwise correlations between the modules in the connectivity measure (kME) of the module eigen-protein are presented in **Figure 3C**, together with lists of module IDs, module colors, and their eigen-proteins representing protein expressions in a module.

**Figure 4:**
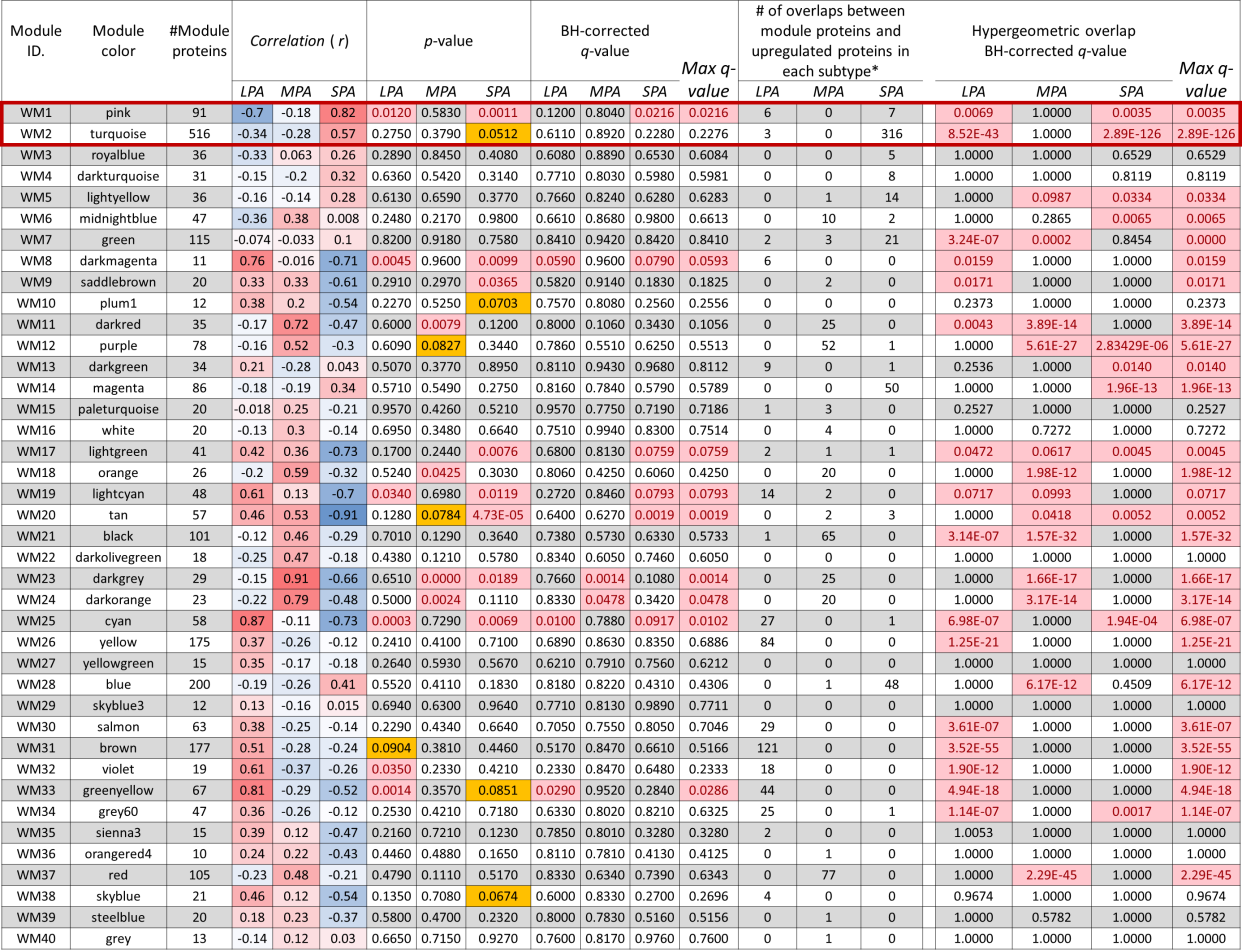
Relationship between module eigen-proteins and the clinical traits of three subtypes LPA, MPA, and SPA. Each row in the embedded table represents weighted gene co-expression network analysis results for each module. The first and second columns in the table represent the module identification and color name of a module, respectively. The third column represents the number of proteins in each module. The table is color-coded by the correlation coefficient, in which the intensity and direction of the correlations are indicated (red, positive correlation; blue, negative correlation). The seventh to ninth columns present the *p*-values of the correlation coefficients, and the tenth to thirteenth columns are *q*-values by multiple testing correction using the Benjamini–Hochberg method, where significant *p*-value (<0.05) and *q*-values (< 0.1) are highlighted in bright red background. *p*-values between 0.05 and 0.1 are highlighted in yellow background. The fourteenth to sixteenth columns present a number of overlaps between module proteins and proteins upregulated in each subtype. The eighteenth to twenty-one columns present results of the statistical over-representative analysis (ORA) using the hypergeometric overlapping test of all the WGCNA modules with proteins upregulated (more than twice) uniquely for the individual subtypes: 397 proteins in LPA, 314 in MPA, and 474 in SPA, where the Benjamini–Hochberg corrected significant *q*-values (< 0.05) are highlighted in bright red background.

Six WGCNA modules were identified with high correlations (*r* > 0.5) and statistical significance (multiple testing correction using the Benjamini–Hochberg method: *q* < 0.05) with clinical traits (**Figure 4**). The WM25 (cyan: *r* = 0.87, *q* = 0.0102) and WM33 (green-yellow: *r* = 0.81, *q* = 0.0286) modules were significantly correlated with the LPA subtype. The WM23 (dark-grey: *r* = 0.91, *q* = 0.014) and WM24 (dark-orange: *r* = 0.94, *q* = 0.0478) modules were significantly correlated with the MPA subtype, and WM1 (pink: *r* = 0.82, *q* = 0.0216) was well correlated with the SPA subtype.

Several other WGCNA modules were also correlated (*r* > 0.5) with one of the three traits. WM8 (dark-magenta: *r* = 0.76), WM19 (light-cyan: *r* = 0.61), and WM32 (violet: *r* = 0.61) correlated with the LPA subtype, WM11 (dark-red: *r* = 0.72) and WM18 (orange: *r* = 0.59) with the MPA subtype, and WM2 (turquoise: *r* = 0.57) with the SPA subtype. However, none of these correlations were significant (*q* > 0.05). Trait correlation analysis often tends to overlook important modules. Statistical over-representative analysis (ORA) may help evaluate potential WGCNA modules concerning overlap with uniquely upregulated proteins for each trait. The numbers of proteins in each subtype more than twice as highly expressed compared with other subtypes were 407 in LPA, 321 in MPA, and 482 in SPA, respectively. Overlap of the WGCNA modules with that of the protein groups was assessed using ORA (**Figure 4**). In this study, we focused on the WGCNA modules, WM1 (pink) and WM2 (turquoise), significantly associated with the high-risk subtype, SPA (**Figure 4**).

### 3.3 Protein-protein interaction networks and functional enrichment

Using the Search Tool for the Retrieval of Interacting Genes/Proteins (STRING) database version 11.5 (https://string-db.org/) (15), human PPI networks were obtained for the WGCNA modules. The PPI networks for the WM1 and WM2 modules associated with the SPA subtype were reconstructed using Cytoscape (version 3.9.1) software (Institute for Systems Biology, Seattle, WA, USA: https://cytoscape.org/) (16) (**Supplementary Figure S2**). Top hub proteins were calculated using the *cytoHubba* plugin with maximal clique centrality (MCC) (17). In these data-driven protein co-expression networks, eigen-proteins are indicated in red letters, hub proteins in red to orange fill colors, and some key proteins are denoted with red borders. Top STRING enrichment results for the WGCNA modules, WM1 and WM2, significant to the SPA subtype are shown in **Supplementary Table S1**.

The functional enrichment obtained for the WM1 module included the following: (i) SRP-dependent co-translational protein targeting to membrane, translational initiation, nuclear-transcribed mRNA catabolic process, nonsense-mediated decay, and protein targeting to membrane as biological processes (GO); and (ii) L13a-mediated translational silencing of ceruloplasmin expression, GTP hydrolysis and joining of the 60S ribosomal subunit, eukaryotic translation termination, and NMD independent of the exon junction complex (EJC) (**Supplementary Table S1**). The eigen-protein RPS4X (small ribosomal subunit protein eS4, also known as SCR10) is a component of the 40S subunit and is involved in L13a-mediated translational silencing of ceruloplasmin expression, eukaryotic translation termination, and NMD independent of the exon junction complex (EJC).

The pathways enriched for the WM2 (turquoise) module included the following: (i) antigen processing and presentation of peptide antigen via MHC class I, NIK/NF-κB signaling, and regulation of transcription from RNA polymerase II promoter in response to hypoxia as biological processes (GO); (ii) regulation of expression of SLITs and ROBOs, cross-presentation of soluble exogenous antigens (endosomes), and NIK to noncanonical NF-κB signaling (**Supplementary Table S1**). The eigen-protein tropomyosin 4 (TPM4) is a member of the tropomyosin family of actin-binding proteins, which is involved in stabilizing cytoskeleton actin filaments.

### 3.4 Semi-quantitative protein expression and multivariate correlation analysis

Forty eigen-proteins together with fifteen key proteins, which were representatively expressed throughout all 40 modules, were subjected to multivariate correlation analysis (MVA), assuming three-group clustering (**Figure 2D**). The eighteen eigen-proteins of the WGCNA modules relatively well correlated (*r* > 0.4) with respective subtypes are denoted in colored filled circles (LPA: red; MPA: blue; SPA: green). Those eighteen eigen-protein expressions were clustered into Cluster *A*, Cluster *B*, and Cluster *C*, which well corresponded to the SPA, LPA, and MPA subtypes, respectively. (**Figure 2D**). Upregulated proteins characteristic of the respective subtypes were also well clustered consistently: HLA-G and HLA-B in SPA; C3, RCC1, and HSPG2 in LPA; MYOF and CRABP2 in MPA.

Zhou et al. (20) performed proteomic analyses between low-risk and high-risk subtypes of early-stage lung adenocarcinomas, where the low-risk LPA subtype group (*n* = 31) and the high-risk subtype group (*n* = 28) consisting of both MPA and SPA and reported several proteins differentially expressed more to the high-risk group. Their results were not well linked to our proteomic observations. Some proteins differentially expressed in their high-risk subtype group, such as DNA replicating licensing factors MCMs, were oppositely upregulated in the low-risk subtype LPA subtype in our study, except that the expression of upregulated P4HA2 (Prolyl-4 hydroxylase subunit alpha-2) was observed in the high-risk subtype MPA (*p* = 0.03). There are intrinsic differences between their approach and ours. They directly used sections of tissues collected immediately after resection for proteomic analysis while we collected only cancerous cells directly from the FFPE tissue sections via laser dissection, and they grouped both MPA and SPA as one high-risk group while we did not group MPA and SPA into one.

### 3.5 Canonical pathways enriched by IPA

**Figure 5:**
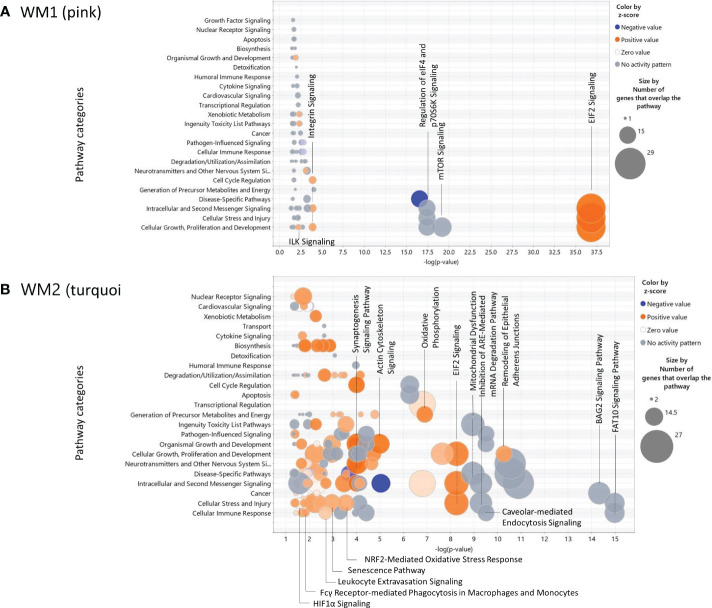
Bubble charts of the canonical pathways significant to the WGCNA modules obtained by IPA. **(A)** the WM1 (pink) network module; **(B)** the WM2 (turquoise) network module.

The canonical pathways and their pathway categories enriched were obtained by the IPA software (http://www.ingenuity.com) (10) for the WM1 and WM2 modules significant to the SPA subtype (**Figure 5**). The WM1 network module was associated predominantly with activated pathways of EIF2 signaling, which are involved in the following pathway categories: Cellular Growth, Proliferation and Development, Cellular Stress and Injury, and Intercellular and Second Messenger Signaling (**Figure 5A**). The WM2 network module was associated with various pathways. The top pathway category was Cellular Immune Response, which included the FAT10 signaling pathway, caveolar-mediated endocytosis signaling, antigen presentation pathway, upregulated leukocyte extravasation signaling, and activated Fcγ receptor-mediated phagocytosis in macrophages and monocytes. The pathway category Cellular Stress and Injury included the FAT10 signaling pathway, inhibition of ARE-mediated mRNA degradation pathway, regulation of eIF4 and p70S6K Signaling, activated pathways of EIF2 signaling, activated NRF2-mediated oxidative stress response, senescence, leukocyte extravasation signaling, hepatic fibrosis signaling, HIF1A signaling, and necroptosis signaling (**Figure 5B**).

### 3.6 Upstream regulators and causal networks enriched by IPA

Upstream regulator and causal network analysis were performed for the WGCNA modules using IPA software (http://www.ingenuity.com) (10). **Table 2** summarizes the top upstream regulators, causal networks, canonical pathways, and diseases or functions predicted for the four WGCNA modules.

**Table 2:** Representative upstream and predicted master regulators (activated or inhibited: *z*-value| ≥ 2.0, and upregulated: 1.5 < *z*-value < 2.0) are summarized for the WGCNA modules, WM1 (pink) and WM2 (turquoise), significantly associated with the SPA subtype.

Module ID (color)	Upstream regulators			Causal networks			Canonical pathways			Diseases or functions		
	Top upstream regulators	*z*-score	*p*-value of overlap	Top master regulators*	*z*-score	*Network bias-corrected p*-value	Top 5 annotations	*z*-score	*p*-value	Top annotations	*z*-score	*p*-value
**WM1 (pink)**	*LARP1* ^a^	-5.10	7.19E-44	*PCGEM1*	4.87	1.00E-04	EIF2 Signaling	3.74	1.58E-37	Viral Infection	3.57	3.17E-08
	*MLXIPL*	4.80	4.16E-31	*MLXIPL*	4.80	0.0001	mTOR Signaling	−	6.31E-20	Infection of cervical cancer cell lines	2.97	0.000324
	*MYCN* ^a^	4.31	1.49E-28	*MYC*	4.77	0.0001	Regulation of eIF4 and p70S6K Signaling	−	3.98E-18	Development of cytoplasm	2.78	0.00000629
	*MYC*	4.87	2.60E-24	*HMGCR*	4.53	0.0001	TCA Cycle II (Eukaryotic)	−	8.91E-05	Fibrogenesis	2.78	0.000388
	*RICTOR*	-4.24	3.80E-17	*CXCL14*	4.36	0.0001	Integrin Signaling	2.45	1.32E-04	Infection of cells	2.78	0.0000202
	*Lh*	4.12	6.30E-17	*MYCN* ^a^	4.32	0.0001				Necrosis	-2.93	5.33E-09
	*DDX3X*	3.13	9.48E-17	*CD8B* ^a^	4.15	0.0001				Cell death of cancer cells	-3.77	6.12E-14
	*TCR* ^a^	2.91	0.0000	*CD8A* ^a^	4.15	0.0001				Cell death of tumor cells	-3.90	1.6E-13
	*RBM20*	-2.12	1.08E-08	*Lh*	4.12	0.0001				Cell death of osteosarcoma cells	-4.00	4.22E-20
	*CD3*	3.16	1.40E-07	*CD3-TCR* ^a^	3.41	0.0001						
	*NFE2L2*	2.97	0.0001	*DDX3X*	3.16	0.0001						
	*HRAS*	2.21	0.0015	*RICTOR*	-4.24	0.0001						
	*NKX2-3*	-2.24	0.0016	*LARP1* ^a^	-5.10	0.0001						
	*TGFB1*	2.44	0.0041	*CD3*	3.16	0.0026						
				*CD37* ^a^	-3.40	0.0041						
				*UNC119* ^a^	4.84	0.0045						
**WM2 (turquoise)**	*MYC*	3.90	5.72E-27	*LARP1*	-4.36	0.0001	Synaptogenesis Signaling Pathway	3.77	9.33E-05	Viral Infection	8.48	2.28E-19
	*RICTOR*	-5.67	9.94E-23	*CLPP*	-4.47	0.0001	Integrin Signaling	3.74	9.77E-05	Infection by RNA virus	7.16	3.29E-16
	*CLPP*	-4.45	2.83E-17	*RICTOR*	-5.78	0.0001	Actin Cytoskeleton Signaling	3.61	1.05E-05	Infection of cells	7.12	1.13E-14
	*IL4*	5.21	2.86E-17	*Cbp/p300* ^c^	5.46	0.0001	EIF2 Signaling	3.32	5.62E-09	Cell viability	6.72	1.28E-11
	*LARP1*	-4.36	4.93E-15	*Hif* ^c^	4.55	0.0001	SNARE Signaling Pathway	3.32	1.10E-04	Cell survival	6.54	7.39E-14
	*TGFB1*	5.45	1.19E-12	*CTNNAL1* ^c^	4.59	0.0001	Oxidative Phosphorylation	3.21	1.26E-07	Cell viability of tumor cell lines	6.15	1.81E-11
	*TEAD1*	4.57	1.36E-11	*Tcrd* ^c^	4.57	0.0002				HIV infection	5.68	2.93E-06
	*NFE2L2*	5.80	1.40E-11	*CTNNβ-LEF1*	4.52	0.0001				Infection by HIV-1	5.41	1.26E-06
	*INSR*	3.80	8.99E-10	*CTNNB1*	4.40	0.0001				Infection of tumor cell lines	5.39	6.95E-09
	*MLXIPL*	4.20	2.39E-09	*IL20RA* ^c^	4.36	0.0002				Infection of cervical cancer cell lines	5.19	3.87E-07
	*IL1B*	3.52	4.60E-09	*NFE2L2*	5.92	0.0003				Cell movement	5.04	8.69E-12
	*ESR2*	3.74	8.05E-09	*FGFR1* ^c^	7.46	0.0005				Organization of cytoplasm	5.04	3.35E-06
	*IFNG^c^*	4.51	1.39E-08	*RAB27A* ^c^	3.94	0.0007				Cell death of osteosarcoma cells	-5.29	1.71E-20
	*TEAD1*	2.12	2.09E-08	*Lilrb2* ^b,c^	-4.24	0.0007				Organismal death	-9.60	2.56E-12
	*RICTOR*	-2.65	2.03E-05	*IGF1R* ^c^	3.88	0.0008						
	*NFkB (complex)*	3.87	8.69E-05	*CD19*	5.81	0.0009						
	*SMARCA4*	3.72	0.0001	*PSMB1* ^c^	-4.07	0.0009						
	*IFN Beta* ^c^	2.07	0.0006	*CD80* ^c^	7.31	0.0186						
	*Interferon alpha^c^*	3.61	0.0022	*CD86* ^c^	6.06	0.0187						
	*LCK ^c^*	3.76	0.0219	*CD80/CD86* ^c^	6.35	0.0438						

Superscripts a), b), and c) denote master and upstream regulators targeting module-member proteins, including the eigen-proteins, PRS4X and TPM4, and HLA-G, respectively.

Highly activated upstream regulators in the WM1 protein networks included MLXIPL (*z* = 4.80, *p*-value of overlap = 4.16 ×10^−31^), MYCN (*z* = 4.12, *p*-value = 6.30 ×10^−17^), MYC (*z* = 4.87, -value = 2.60 ×10^−24^), Lh (*z* = 4.87, *p*-value = 6.30 ×10^−17^), DDX3X (*z* = 3.13, *p*-value = 9.48 ×10^−17^), TCR (*z* = 2.91, *p*-value = 1.94 ×10^−10^), CD3 (*z* = 3.16, *p*-value = 1.40 ×10^−7^), NFE2L2 (*z* = 2.97, *p*-value = 0.0001), and both CD8A and CD8B (*z* = 4.15, *p*-value = 0.0001), whereas highly inhibited regulators included LARP1 (*z* = −5.10, *p*-value = 7.19 ×10^−44^) and RICTOR (*z* = −4.24, *p*-value = 3.80 ×10^−17^) (**Table 2**). Molecules in the WM1 module targeted by LARP1, MYCN, and TCR included the eigen-protein RPS4X. The carbohydrate-responsive element-binding protein (ChREBP) encoded by *MLXIPL* is a basic helix-loop-helix leucine zipper transcription factor of the MYC/MAX/MAD superfamily. Its activation promotes cancer cell proliferation and inhibits cancer cell apoptosis by promoting aerobic glycolysis in various cancers (21).

CD3, CD8A, CD8B, and TCR (T-cell receptor) are drivers of the immune system and the immune defense against intracellular pathogens, such as viruses and bacteria, as well as tumors. CD8 is expressed on the surface of cytotoxic T lymphocytes (CTLs), dendritic cells, macrophages, monocytes, and NK cells and exists as either a homodimer (two CD8A chains) or heterodimer (CD8A and CD8B chains), which are co-receptors of the TCR-CD3 complex and binds to MHC-I to activate T cells. CD8A recruits T-cell-specific protein tyrosine kinase (LCK) or linker for activation of T-cells family member 1 (LAT) to activate downstream signaling pathways, whereas CD8B increases the avidity of the CD8/MHC/TCR complex to increase IL-2 production.

Highly inhibited La-related protein 1 (LARP1), the master regulator of the cap-dependent top mRNA translation, strongly suggests the inhibition of protein synthesis via cap-dependent mRNA translation or the activation of the cap-independent, IRES-mediated translation of mRNA subsets encoding oncogenic proteins, such as HIF1A, and MYC (22).

Regarding the WM2 module, highly activated upstream regulators included MYC, NFE2L2, MLXIPL, IFNG (*z* = 4.51, *p*-value of overlap = 1.39 ×10^−8^), NFκB (complex) (*z* = 3.87, *p* = 8.69 ×10^−5^), FGFR1 (*z* = 7.46, network bias-corrected *p* = 0.0005), TCRD (T-cell receptor delta chain) (*z* = 4.57, *p* = 0.0001), CTNNβ-LEF1 (*z* = 4.52, *p* = 0.0001), CTNNβ1 (z = 4.40, p = 0.0001), CD80 (*z* = 7.31, *p* = 0.019), CD86 (*z* = 6.06, *p* = 0.019), hypoxia-induced factors *Hif* (*z* = 4.55, *p* = 0.0001), and LCK (*z* = 3.76, *p* = 0.0219), whereas highly inhibited proteins included LARP1 and RICTOR, which were common with the WM1 module, and CLPP (*z* = −4.47, *p* = 0.0001) and LILRB2 (*z* = −4.24, *p* = 0.0007) (**Table 2**).

FGFR1 is a cell-surface receptor for fibroblast growth factors that regulate embryonic development, cell proliferation, differentiation, and migration. Its amplification has been frequently reported in NSCLC (23). Activation of CTNNβ1 and CTNNβ-LEF1 implied activation of Wnt/β-catenin signaling in which β-catenin is stabilized and accumulated as free β-catenin in the cytosol and is subsequently translocated into the nucleus and activates the TCF (T-cell factor)/LEF (lymphoid enhancer factor)-dependent transcription of Wnt target genes, key factors in cell proliferation and invasion (24, 25). Proto-oncogene *LCK* encodes lymphocyte-specific protein tyrosine kinase LCK, an Src family tyrosine kinase, which plays an important role in TCR-linked signal transduction. CD28-induced LCK activation is an important mediator of T-cell activation. Recently, mathematical models for LCK autophosphorylation suggest that LCK is involved in the early stages of T-cell activation and response derived from PD-1 signaling suppression (26). It also potentially mediates PD-1-induced inhibition of early TCR signaling (27), which is important to cancer immune checkpoint therapy. LILRB2 is Leukocyte immunoglobulin-like receptor subfamily B member 2 (also known as ILT-4, immunoglobulin-like transcript 4) is a tumor immune checkpoint molecule. Caseinolytic protease P (CLPP) is involved in the mitochondrial unfolded protein response and cellular bioenergetics (28).

Interestingly, all of *Hif* (complex), TRCD, FGFR1, IFNG, CD80, CD86, LCK, and LILRB2 target molecules including HLA-G in the WM2 module, whereas LILRB2 targets module molecules including both HLA-G and eigen-protein TPM4 (**Table 2**). Schreiber et al. demonstrated that the effects of IFNG are generally pro-tumorigenic during the immune escape stage of cancer immunoediting, in which IFNG increases inhibitory immune checkpoint molecules, including HLA-G, to promote the formation of a tolerant immune microenvironment (29).

HLA-G exerts inhibitory effects on both innate and adaptive effectors through direct binding to inhibitory receptors, such as immunoglobulin-like transcripts, LILRB1 (also known as ILT-2), and LILRB2, with high affinity compared with other HLA class I molecules. HLA-G can inhibit many players associated with the anti-tumor response throughout early and late tumor stages, although CTLA-4 and PD-1 are predominantly expressed in higher tumor grades (30, 31). The networks associated with LILRB2 inhibition include the activated hub regulator, toll-like receptor 4 (TLR4, also known as CD284). TLR signaling activates various signaling molecules, including nuclear factor κB (NFκB), extracellular signal-regulated kinase (ERK/JNK/p38), and induces the synthesis of immunologic factors including IL-6, IL-12, PD-L1, and HLA-G. This results in the resistance of tumor cells to CTL attack and tumor cell immune evasion (32). Highly expressed HLA-G and highly inhibited LILRB2 implicated involvement of immune tolerance or anti-tumor immune escape. The volcano plots revealed high expression of HLA-G associated with the SPA subtype (**Figures 2A–C**). A web-based survival analysis (KMplot) for mRNA data of lung adenocarcinoma (*n*  =  719) indicated that high HLA-G expression was significantly associated with poor OS (log-rank test *p*  =  1.0 × 10^−11^; hazard ratio: 3.1 (2.2–4.36)) (https://kmplot.com/analysis/) (**Supplementary Figure S3**) (33). Yan et al. interestingly showed a predominant expression of soluble HLA-G (sHLA-G) in tumor cells not from squamous cell carcinoma but adenocarcinoma of lung (34).

### 3.7 Genomic alterations of LPA, MPA, and SPA based on the MSKCC database

Lung adenocarcinoma genomic data from the MSKCC database (Stage I to III: *n*  =  604) was analyzed for prognostic markers associated with the predominant histologic subtypes in lung adenocarcinoma using the Memorial Sloan Kettering–Integrated Mutation Profiling of Actionable Cancer Targets (MSK-IMPACT) platform (5). We extracted data for the LPA (*n* = 88), MPA (*n* = 37), and SPA (*n* = 68) subtypes, and the genomic alteration profiles were visualized using the cBioPortal for Cancer Genomics (https://www.cbioportal.org/) (**Supplementary Figure S4**). The oncoprints showed genomic alterations characteristic of the three subtypes as follows: LPA, TP53 (17%), EGFR (43%), and KRAS (33%), whereas EGFR and KRAS were mutually exclusive (*q* < 0.001); MPA, TP53 (41%), EGFR (22%), and KRAS (32%); SPA, TP53 (75%), which was the highest among the three subtypes, EGFR (22%), and KRAS (38%).

In connection with the above genomic alterations, upregulated expressions of HLA I class molecules such as HLA-G and HLA-B were observed in proteomic analysis. (**Figures 2A, B**). It has been reported that there is a positive correlation between mutational burden and the expression of most HLA class I molecules (35, 36), and HLA class I molecules are associated with the innate immune response as ligands of inhibitory killer cell immunoglobulin-like receptors (KIRs) of Natural Killer (NK) cells (37).

## 4 Discussion

The WGCNA analysis was successfully applied to MS-based proteomic data. Forty co-expression network modules were identified, among which two network modules were significantly associated with solid predominant adenocarcinoma, SPA (**Figure 4**). Volcano plots of the proteins revealed high expression levels of HLA-G (human leukocyte antigen G) in the SPA subtype (**Figures 2A, B**). A multivariate correlation analysis (MVA) of eigen-proteins mostly grouped into Cluster *A*, *B*, and *D*, which correspond well to the SPA, MPA, and LPA subtypes, respectively (**Figure 2C**). Oncoprint analysis of three subtypes exhibited that SPA interestingly exhibited a highly frequent p53 alteration of 75% although MPA and LPA harbored a frequency of p53 alterations, 41% and 17%, respectively (**Supplementary Figure S4**). High TP53 alteration seems to reflect a high malignancy of SPA.

Upstream analysis using IPA predicted that the highly activated oncogenic regulators, MYC, MLXIPL, and MYCN may reflect an aberrant feature of the SPA subtype, that activation of the redox master regulator NFE2L2 indicates an occurrence of oncogenic signaling in response to oxidative stresses and that both the highly inhibited LARP1 and activated pathways of EIF2 signaling indicated a switch to IRES-mediated mRNA translation, resulting in the generation of oncogenic proteins, such as hypoxia-inducible factors (*Hif*). Activation of Wnt/β-catenin signaling also might participate in the disease-related interaction networks of SPA, which results in Wnt target gene products by the TCF/LEF-dependent transcription.

**Figure 6:**
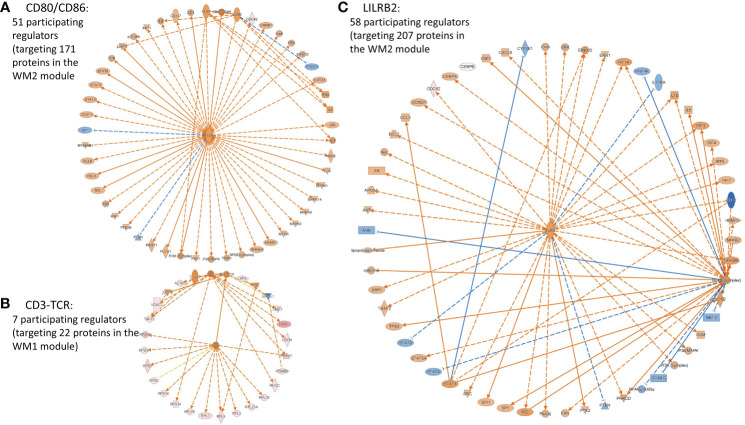
Representative cancer cell immunity-related causal networks of **(A)** CD80/CD86, **(B)** CD-TCR, and **(C)** LILBR2, associated with the SPA subtype, predicted by IPA. Node shapes indicate molecular types: triangle, kinase; square (dashed), growth factor; rectangle (horizontal), ligand-dependent nuclear receptor; rectangle (vertical), ion channel; diamond (vertical), enzyme; diamond (horizontal), peptidase; trapezoid, transporter; oval (horizontal), transcription regulator; oval (vertical), transmembrane receptor; double circle, complex; and circle, other. Orange/light orange and blue/light blue colors indicate the extent of confidence for predicted activation and inhibition, respectively. Lines denote predicted relationships. A solid or dashed line indicates direct or indirect interaction, respectively. Orange, leading to activation; blue, leading to inhibition; yellow, findings inconsistent with the state of a downstream molecule; grey, not predicted effect. CD80/CD86, T-lymphocyte activation antigens CD80/CD86 (Activation B7-1/B7-2 antigens); CD3-TCR, CD3, TCR (T cell receptor complex); LILRB2, Leukocyte immunoglobulin-like receptor subfamily B member 2, also known as Immunoglobulin-like transcript 4 (ILT-4).

Interestingly, SPA-significant co-expression network modules, WM1 and WM2, also involved highly activated IFNG, TCRD, CD3-TCR, CD8A, CD8B, CD3, CD80/CD86, and highly inhibited LILRB2, all of which are associated with the adaptive immune system and defense, and importantly which all target module molecules including the HLA class I molecule HLA-G. Representative immune system-related causal networks associated with the SPA subtype are presented in **Figure 6**. Moreover, our observation of the immune checkpoint molecule, HLA-G, significantly upregulated in the SPA subtype and its counterpart LILRB2 predicted to be significant and highly inhibited implicate the involvement of the immune tolerance or anti-tumor immune escape processes in early-stage SPA, although CTLA-4 and PD-1 may not yet be expressed at early-stage.

The Applied Proteogenomic Organizational Learning and Outcomes (APOLLO) research network (38) conducted the deep proteogenomic profiling of 87 lung adenocarcinoma tumors from a cohort of individuals in the United States, which utilizes the integrative approaches, including whole-genome, transcriptome, and MS-based proteomic and phosphoproteomic sequencing, and in which adenocarcinoma tumor subtypes were stratified according to RNA expression subtypes, such as terminal respiratory unit (TRU), proximal proliferative (PP), and proximal inflammatory (PI). They reported that PI enriched with solid predominant histological subtype (SPA) overexpressed the immune cell marker clusters, and its network is characteristically associated with enhanced IFNG signaling and inflammation, high tumor mutational burden (TMB), coupled with PD-L1 protein and CTLA4 RNA expression, and suggested that the PI subtype may have the subset of tumors most likely to respond to immune checkpoint inhibitors (38).

Recently, Li et al. (39) conducted a multi-omics analysis of 1,078 untreated lung adenocarcinoma patients with clinicopathologic, genomic, transcriptomic, and proteomic data from public and internal cohorts. SPA had molecular features including significantly higher tumor mutation burden (TMB), the higher frequency of TP53 mutation together with EGFR/TP53 co-mutation, and higher immuno-resistant microenvironment, that indicates a poor response to chemotherapy, such as tyrosine kinase inhibitors (TKIs). Indeed, SPA showed upregulated expression of immunotherapy-related genes relevant to strong immunogenicity. Moreover, it was shown in the cohort of lung adenocarcinoma patients who received neoadjuvant immunotherapy that SPA relatively well responded to immunotherapy. They concluded that SPA would be more suitable for immunotherapy while less suitable for chemo- and targeted therapy (39).

Both studies (38, 39) seem to consistently reflect the molecular features of SPA and give corroboration to our findings in this study. One limitation of this study was the number of patients examined because of the limited number of early-stage cases pathologically confirmed and available for proteomic analysis in our hospital.

## 5 Conclusion

In summary, we successfully identified disease-related co-expression protein networks by WGCNA analysis applied to the proteomic datasets, following MS-based proteomic analysis of cancerous cells laser microdissected from FFPE tissue specimens of three lung adenocarcinoma subtypes, LPA, MPA, and SPA. Upstream regulator and causal network analysis performed for protein co-expression networks significantly associated with the SPA subtype implicated not only oncogenic signalings including cap-independent IRES-dependent mRNA translation and NRF2-mediated oxidative stress response but also highly activated molecular networks relating to the adaptive immune system and immune tolerance more likely associated with malignancies characterizing the SPA subtype, together with its high frequency of TP53 mutation. Although the number of patient samples examined was limited in this study, we are planning a larger cohort study of patient-derived samples, including genomic alteration analysis to investigate core data-driven proteogenomic networks. This approach will provide clinically important information on proteogenomic landscapes of the high-risk adenocarcinoma subtype SPA of the lung.

## Data availability statement

The datasets presented in this study can be found in online repositories. The names of the repository/repositories and accession number(s) can be found below: http://www.proteomexchange.org/, PXD037831.

## Ethics statement

The studies involving human participants were reviewed and approved by the Institutional Review Board of St. Marianna University School of Medicine (approval no. 1461). Written informed consent to participate in this study was provided by the patient/participants or the patient/participants’ legal guardian/next of kin. FFPE tumor tissue blocks from 12 surgical specimens histologically confirmed as lung LPA, MPA, and SPA were obtained without patient identifiers from the St. Marianna University School of Medicine Hospital.

## Author contributions

TN: Conceptualization, Data curation, Investigation, Methodology, Project administration, Software, Writing – original draft, Writing – review & editing. ÁV: Conceptualization, Data curation, Project administration, Writing – review & editing. HN: Conceptualization, Project administration, Writing – review & editing. KF: Formal analysis, Writing – review & editing. SN: Resources, Writing – review & editing. NF: Supervision, Writing – review & editing. HSak: Writing – review & editing, Resources. HSaj: Conceptualization, Project administration, Writing – review & editing.

## References

[1] WHO classification of tumours editorial board. In: Thoracic Tumours, 5th ed. International Agency for Research on Cancer, Lyon, France.

[2] Yuan Y Ma G Zhang Y Chen H. Presence of micropapillary and solid patterns are associated with nodal upstaging and unfavorable prognosis among patient with cT1N0M0 lung adenocarcinoma: a large-scale analysis. J Cancer Res Clin Oncol (2018) 144:743–9. doi: 10.1007/s00432-017-2571-7 PMC11813456 29392402

[3] Yoshiya T Mimae T Tsutani Y Tsubokawa N Sasada S Miyata Y. Prognostic role of subtype classification in small-sized pathologic N0 invasive lung adenocarcinoma. Ann Thorac Surg (2016) 102:1668–73. doi: 10.1016/j.athoracsur.2016.04.087 27344277

[4] Cao Y Zhu LZ Jiang MJ Yuan Y. Clinical impacts of a micropapillary pattern in lung adenocarcinoma: a review. Onco Targets Ther (2015) 9:149–58. doi: 10.2147/OTT.S94747 PMC4706128 26770064

[5] Caso R Sanchez-Vega F Tan KS Mastrogiacomo B Zhou J Jones GD. The underlying tumor genomics of predominant histologic subtypes in lung adenocarcinoma. J Thorac Oncol (2020) 15:1844–56. doi: 10.1016/j.jtho.2020.08.005 PMC7704768 32791233

[6] Zhao Y Wang R Shen X Pan Y Cheng C Li Y. Minor components of micropapillary and solid subtypes in lung adenocarcinoma are predictors of lymph node metastasis and poor prognosis. Ann Surg Oncol (2016) 23:2099–105. doi: 10.1245/s10434-015-5043-9 PMC4858562 26842488

[7] Jeon HW Kim YD Sim SB Moon MH. Comparison of clinical results between high grade patterns in stage I lung adenocarcinoma. Thorac Cancer (2022) 13:2473–9. doi: 10.1111/1759-7714.14578 PMC9436686 35820717

[8] Ujiie H Kadota K Chaft JE Buitrago D Sima CS Lee MC. Solid predominant histologic subtype in resected stage I lung adenocarcinoma is an independent predictor of early, extrathoracic, multisite recurrence and of poor postrecurrence survival. J Clin Oncol (2015) 33:2877–84. doi: 10.1200/JCO.2015.60.9818 PMC4554749 26261257

[9] Nishimura T Nakamura H Végvári Á Marko-Varga G Furuya N Saji H. Current status of clinical proteogenomics in lung cancer. Expert Rev Proteomics (2019) 16:761–72. doi: 10.1080/14789450.2019.1654861 31402712

[10] Krämer A Green J Pollard J Jr Tugendreich S. Causal analysis approaches in Ingenuity Pathway Analysis. Bioinformatics (2014) 30:523–30. doi: 10.1093/bioinformatics/btt703 PMC3928520 24336805

[11] Langfelder P Horvath S. WGCNA: an R package for weighted correlation network analysis. BMC Bioinf (2008) 9:559. doi: 10.1186/1471-2105-9-559 PMC2631488 19114008

[12] Travis WD Brambilla E Nicholson AG Yatabe Y Austin JHM Beasley MB. The 2015 World Health Organization classification of lung tumors: impact of genetic, clinical and radiologic advances since the 2004 classification. J Thorac Oncol (2015) 10:1243–60. doi: 10.1097/JTO.0000000000000630 26291008

[13] Prieto DA Hood BL Darfler MM Guiel TG Lucas DA Conrads TP. Liquid Tissue: proteomic profiling of formalin-fixed tissues. Biotechniques (2005) Suppl:32–5. doi: 10.2144/05386su06 16528915

[14] Carvalho PC Lima DB Leprevost FV Santos MD Fischer JS Aquino PF. Integrated analysis of shotgun proteomic data with PatternLab for proteomics 4.0. Nat Protoc (2016) 11:102–17. doi: 10.1038/nprot.2015.133 PMC5722229 26658470

[15] Szklarczyk D Gable AL Nastou KC Lyon D Kirsch R Pyysalo S. The STRING database in 2021: customizable protein-protein networks, and functional characterization of user-uploaded gene/measurement sets. Nucleic Acids Res (2021) 49:D605–12. doi: 10.1093/nar/gkaa1074 PMC7779004 33237311

[16] Doncheva NT Morris JH Gorodkin J Jensen LJ. Cytoscape stringApp: network analysis and visualization of proteomics data. J Proteome Res (2019) 18:623–32. doi: 10.1021/acs.jproteome.8b00702 PMC6800166 30450911

[17] Chin CH Chen SH Wu HH Ho CW Ko MT Lin CY. cytoHubba: identifying hub objects and sub-networks from complex interactome. BMC Syst Biol (2014) 8 Suppl 4:S11. doi: 10.1186/1752-0509-8-S4-S11 25521941 PMC4290687

[18] Khan A Mathelier A. Intervene: a tool for intersection and visualization of multiple gene or genomic region sets. BMC Bioinf (2017) 18:287. doi: 10.1186/s12859-017-1708-7 PMC5452382 28569135

[19] Thomas PD Ebert D Muruganujan A Mushayahama T Albou LP Mi H. PANTHER: Making genome-scale phylogenetics accessible to all. Protein Sci (2022) 31:8–22. doi: 10.1002/pro.4218 34717010 PMC8740835

[20] Zhou J Liu B Li Z Li Y Chen X Ma Y. Proteomic analyses identify differentially expressed proteins and pathways between low-risk and high-risk subtypes of early-stage lung adenocarcinoma and their prognostic impacts. Mol Cell Proteomic (2021) 20:100015. doi: 10.1074/mcp.RA120.002384 PMC7950210 33508502

[21] Iizuka K Takao K Yabe D. ChREBP-mediated regulation of lipid metabolism: involvement of the gut microbiota, liver, and adipose tissue. Front Endocrinol (Lausanne) (2020) 11:587189. doi: 10.3389/fendo.2020.587189 33343508 PMC7744659

[22] Silvera D Formenti SC Schneider RJ. Translational control in cancer. Nat Rev Cancer (2010) 10:254–66. doi: 10.1038/nrc2824 20332778

[23] Bogatyrova O Mattsson JSM Ross EM Sanderson MP Backman M Botling J. FGFR1 overexpression in non-small cell lung cancer is mediated by genetic and epigenetic mechanisms and is a determinant of FGFR1 inhibitor response. Eur J Cancer (2021) 151:136–49. doi: 10.1016/j.ejca.2021.04.005 33984662

[24] Marie PJ Haÿ E. Cadherins and Wnt signalling: a functional link controlling bone formation. Bonekey Rep (2013) 2:330. doi: 10.1038/bonekey.2013.64 24422077 PMC3722765

[25] Osuka S Zhu D Zhang Z Li C Stackhouse CT Sampetrean O. N-cadherin upregulation mediates adaptive radioresistance in glioblastoma. J Clin Invest (2021) 131:e136098. doi: 10.1172/JCI136098 33720050 PMC7954595

[26] Kreusser LM Rendall AD. Autophosphorylation and the dynamics of the activation of lck. Bull Math Biol (2021) 83:64. doi: 10.1007/s11538-021-00900-9 33932170 PMC8088428

[27] Arulraj T Barik D. Mathematical modeling identifies Lck as a potential mediator for PD-1 induced inhibition of early TCR signaling. PloS One (2018) 13:e0206232. doi: 10.1371/journal.pone.0206232 30356330 PMC6200280

[28] Cormio A Sanguedolce F Pesce V Musicco C. Mitochondrial caseinolytic protease P: A possible novel prognostic marker and therapeutic target in cancer. Int J Mol Sci (2021) 22:6228. doi: 10.3390/ijms22126228 34207660 PMC8228031

[29] Alspach E Lussier DM Schreiber RD. Interferon γ and its important roles in promoting and inhibiting spontaneous and therapeutic cancer immunity. Cold Spring Harb Perspect Biol (2019) 11:a028480. doi: 10.1101/cshperspect.a028480 29661791 PMC6396335

[30] Carosella ED Ploussard G LeMaoult J Desgrandchamps F. A systematic review of immunotherapy in urologic cancer: evolving roles for targeting of CTLA-4, PD-1/PD-L1, and HLA-G. Eur Urol (2015) 68:267–79. doi: 10.1016/j.eururo.2015.02.032 25824720

[31] Carosella ED Gregori S Tronik-Le Roux D. HLA-G/LILRBs: A cancer immunotherapy challenge. Trends Cancer (2021) 7:389–92. doi: 10.1016/j.trecan.2021.01.004 33563576

[32] Huang B Zhao J Li H He KL Chen Y Chen SH. Toll-like receptors on tumor cells facilitate evasion of immune surveillance. Cancer Res (2005) 65:5009–14. doi: 10.1158/0008-5472.CAN-05-0784 15958541

[33] Lánczky A Győrffy B. Web-based survival analysis tool tailored for medical research (KMplot): development and implementation. J Med Internet Res (2021) 23:e27633. doi: 10.2196/27633 34309564 PMC8367126

[34] Yan WH Lu HY Li YY Zhang X Lin A. Significance of tumour cell HLA-G5/-G6 isoform expression in discrimination for adenocarcinoma from squamous cell carcinoma in lung cancer patients. J Cell Mol Med (2015) 19:778–85. doi: 10.1111/jcmm.12400 PMC4395192 25689063

[35] Noblejas-López MDM Nieto-Jiménez C Morcillo García S Pérez-Peña J Nuncia- Cantarero M Andrés-Pretel F. Expression of MHC class I, HLA-A, and HLA-B identifies immune-activated breast tumors with favorable outcome. Oncoimmunology (2019) 8:e1629780. doi: 10.1080/2162402X.2019.1629780 31646075 PMC6791424

[36] Chowell D Morris LGT Grigg CM Weber JK Samstein RM Makarov V. Patient HLA class I genotype influences cancer response to checkpoint blockade immunotherapy. Science (2018) 359:582–7. doi: 10.1126/science.aao4572 PMC6057471 29217585

[37] Ajitkumar P Geier SS Kesari KV Borriello F Nakagawa M Bluestone JA. Evidence that multiple residues on both the alpha-helices of the class I MHC molecule are simultaneously recognized by the T cell receptor. Cell (1988) 54:47–56. doi: 10.1016/0092-8674(88)90178-X 3260136

[38] Soltis AR Bateman NW Liu J Nguyen T Franks TJ Zhang X. Proteogenomic analysis of lung adenocarcinoma reveals tumor heterogeneity, survival determinants, and therapeutically relevant pathways. Cell Rep Med (2022) 3:100819. doi: 10.1016/j.xcrm.2022.100819 36384096 PMC9729884

[39] Li F Wang S Wang Y Lv Z Jin D Yi H. Multi-omics analysis unravels the underlying mechanisms of poor prognosis and differential therapeutic responses of solid predominant lung adenocarcinoma. Front Immunol (2023) 14:1101649. doi: 10.3389/fimmu.2023.1101649 36845145 PMC9946976

