# Supplementary material for: Cancer cell immunity-related protein co-expression networks are associated with early-stage solid-predominant lung adenocarcinoma
Source: Front Oncol. 2024 Feb 21;14:1273780. doi: 10.3389/fonc.2024.1273780 (PMC10915646; doi:10.3389/fonc.2024.1273780)
Supplement: Supplementary file 1 [file DataSheet_1.pdf]

Supplementary Information File 1

Cancer cell immunity-related protein co-expression networks are associated with early-stage solid-predominant lung adenocarcinoma

Toshihide Nishimura, Ákos Végvári, Haruhiko Nakamura, Kiyonaga Fujii, Hiroki Sakai, Saeko Naruki, Naoki Furuya, Hisashi Saji

Figure S1. Gene ontology analysis of identified proteins.

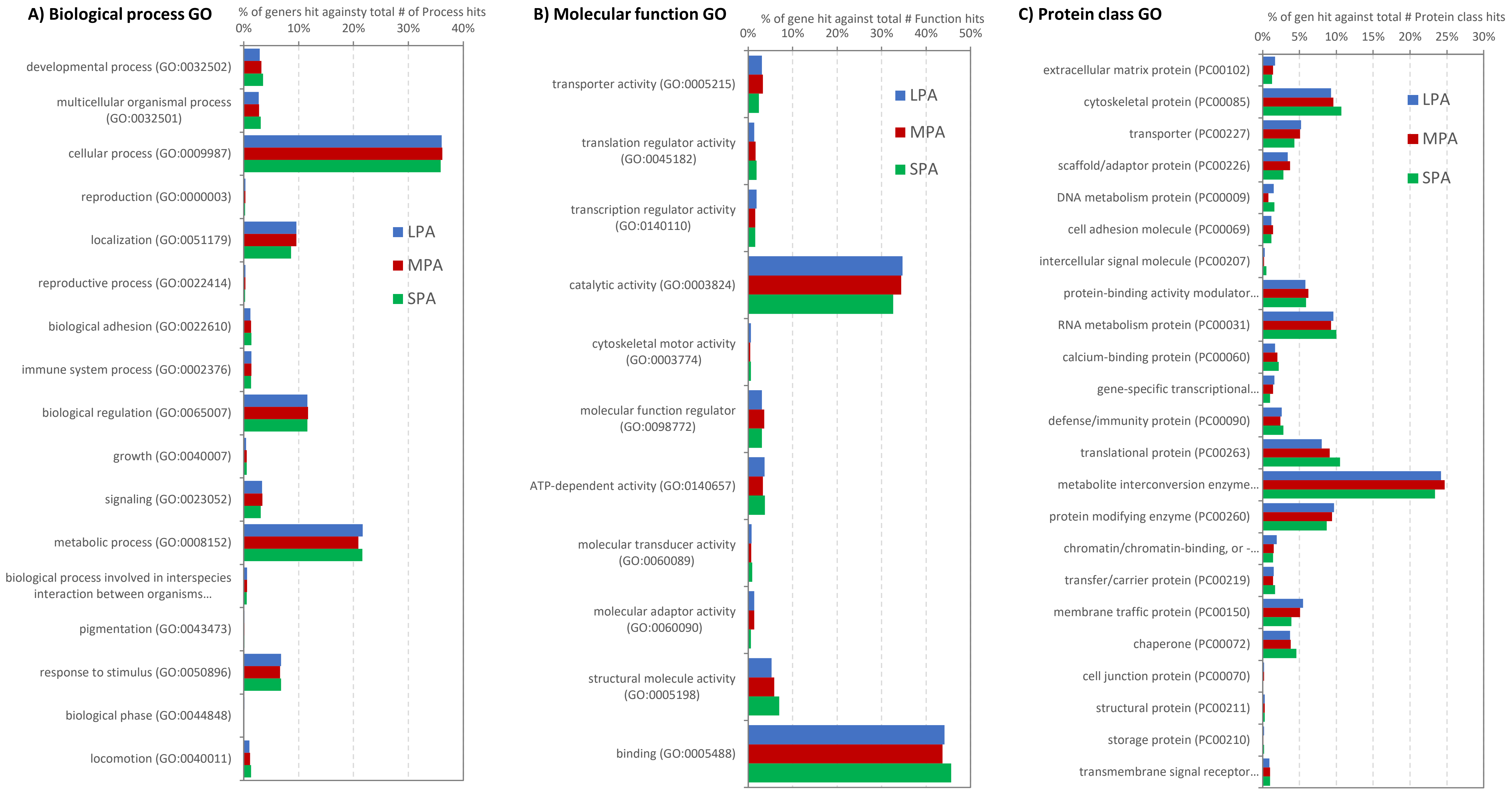

Figure S2. Data-driven protein co-expression networks characteristic of the lung solid adenocarcinoma subtype SPA (WM1 and WM2). Circle nodes with a red letter denote the WGCNA eigen-proteins and those with a fill color ranging from red to orange indicate top high to lower rank hub proteins, according to the maximal clique centrality (MCC) calculated in *cytoHubba*.

A. WM1 (pink)

Eigen-protein: RPS4X

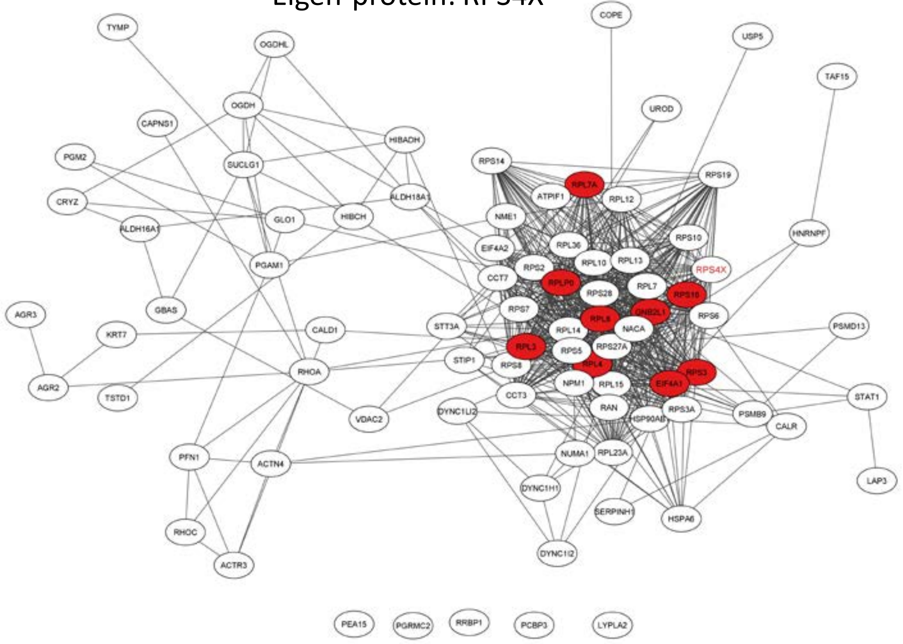

B. WM2 (turquoise)

Eigen-protein: TPM4

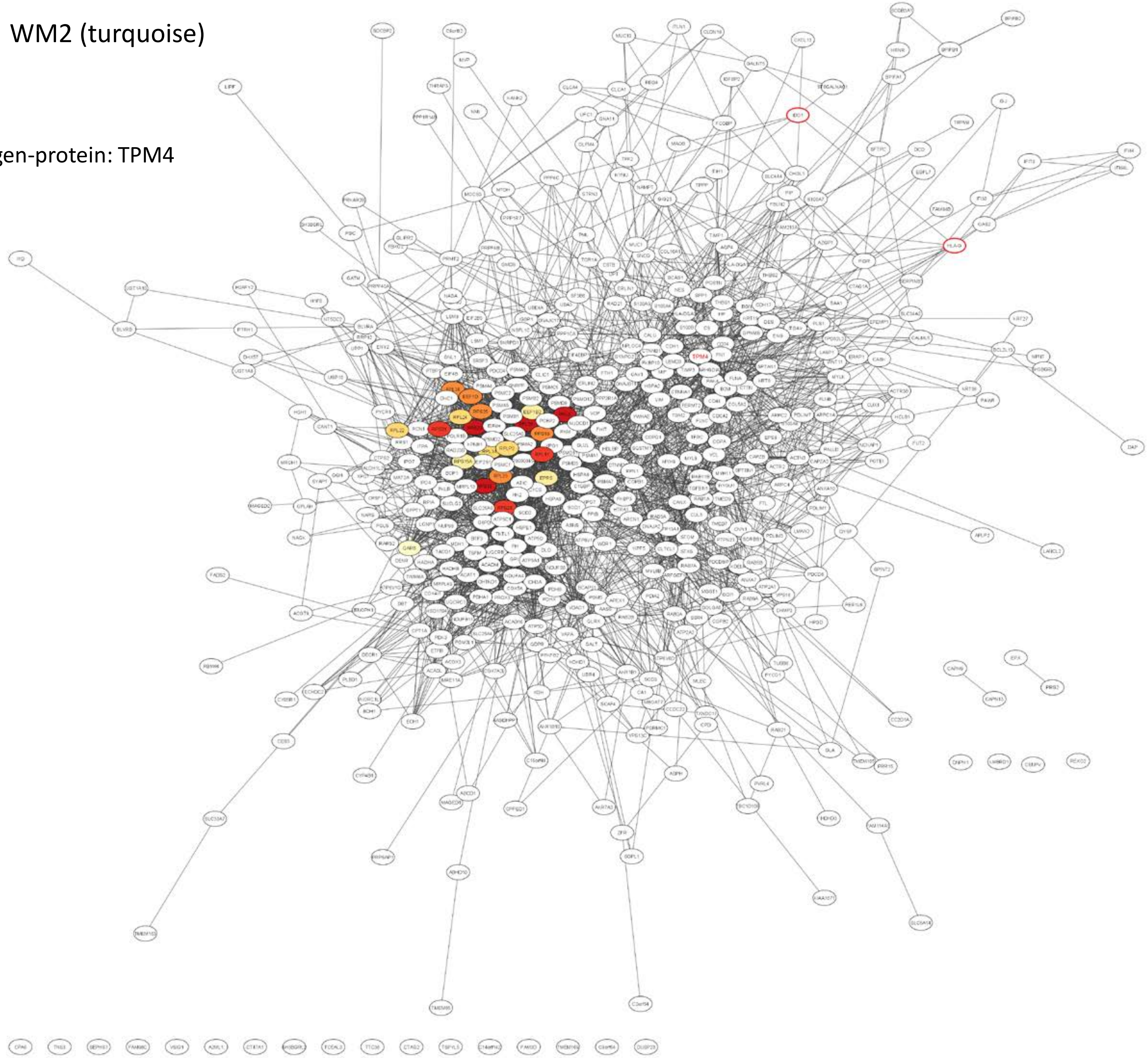

Figure S3. The Kaplan-Meier plots for HLA-G.

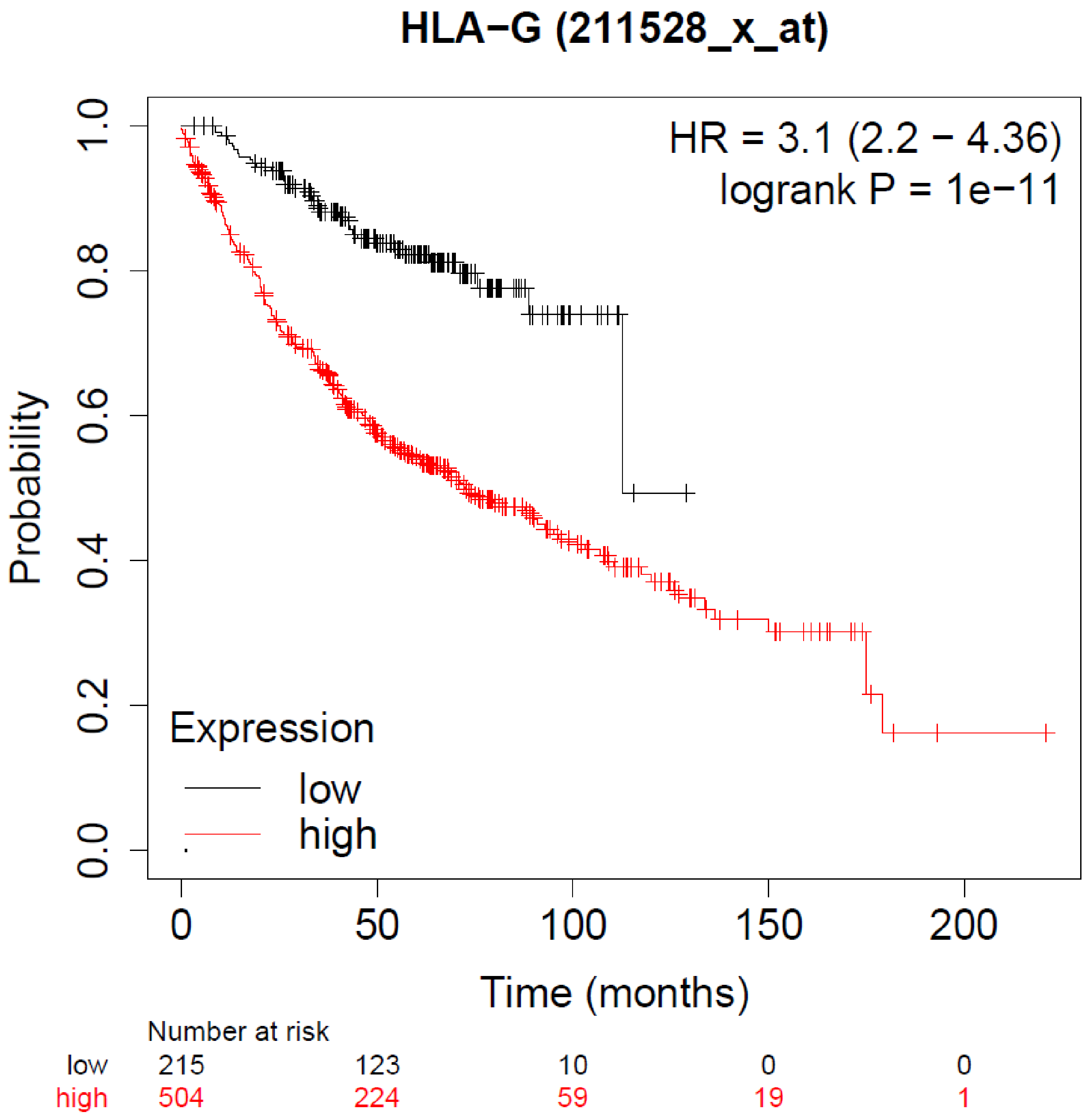

Figure S4. Genomic alteration landscape of early-stage LPA, MPA. And SPA, obtained from lung adenocarcinoma (MSKCC, 2020) database: *n* = 604). The percentage of samples with genomic alterations is presented at the left of each subtype column.

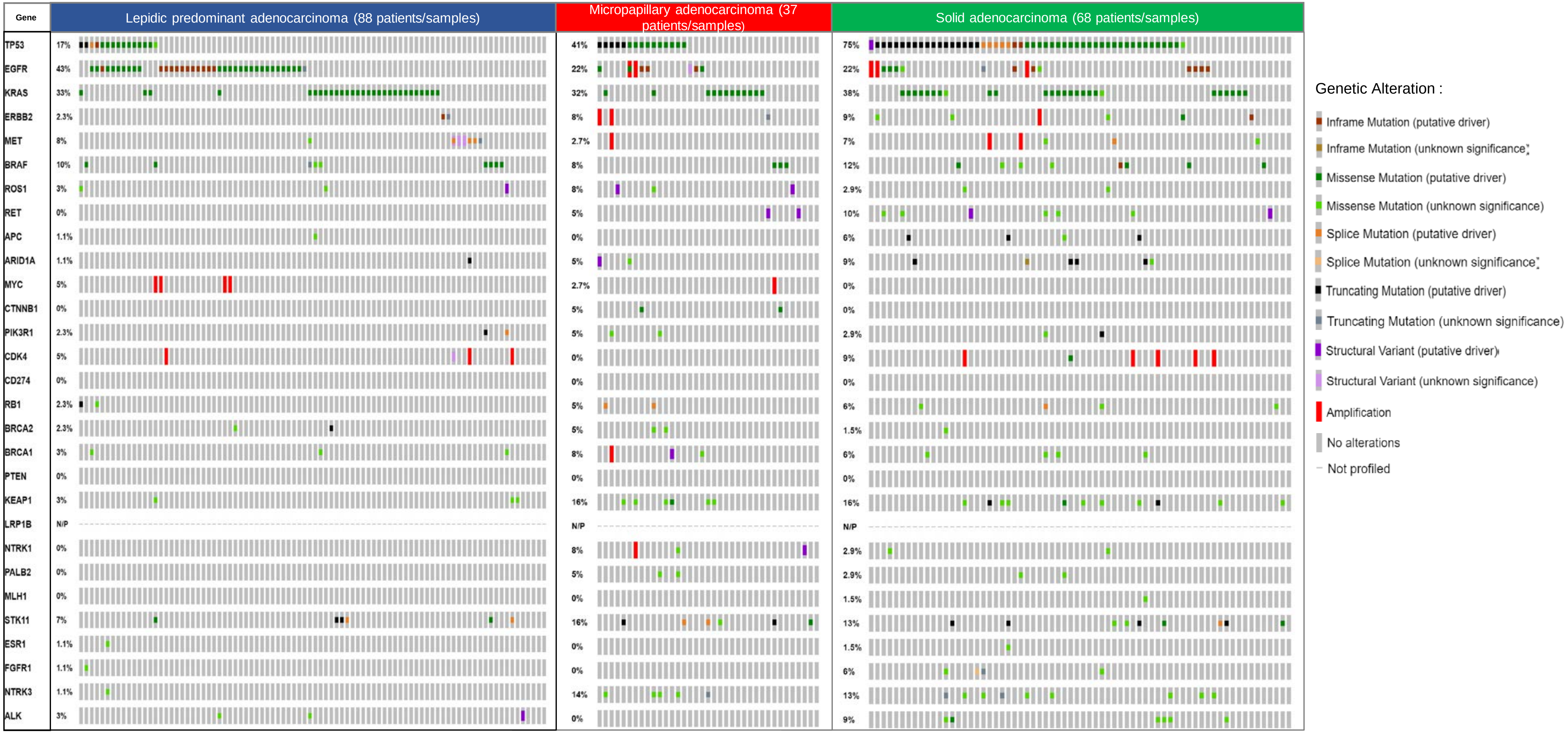

Lung Adenocarcinoma (MSKCC, 2020): Targeted sequencing of 604 lung adenocarcinoma tumor/normal pairs via MSK-IMPACT (https://datacatalog.mskcc.org/dataset/10587)

Table S1. The results of STRING functional enrichment analysis for the six WGCNA modules significant to the SPA subtype. top 20 pathways of biological process (GO) and Reactome pathways with a strength higher than 0.8 are presented in the order of significance by FDR. Strength (Log10 (observed/expected)) is a measure of the enrichment effect, that is the ratio between i) the number of proteins in a network that is annotated with a term and ii) the number of proteins expected to be annotated with this term in a random network of the same size.

Module ID (color)	(term ID) term description	observed gene count	background gene count	strength	false discovery rate
	GO Biological process				
WM1 (pink)	(GO:0006614) SRP-dependent cotranslational protein targeting to membrane	27	96	1.81	2.33E-35
	(GO:0006413) Translational initiation	29	141	1.67	3.03E-35
	(GO:0019083) Viral transcription	27	115	1.73	2.76E-34
	(GO:0000184) Nuclear-transcribed mrna catabolic process, nonsense-mediated decay	27	119	1.71	5.35E-34
	(GO:0006612) Protein targeting to membrane	28	165	1.59	2.12E-32
	(GO:0006412) Translation	32	366	1.3	3.18E-29
	(GO:0016032) Viral process	38	776	1.05	5.57E-27
	(GO:0006518) Peptide metabolic process	33	503	1.17	1.17E-26
	(GO:0034655) Nucleobase-containing compound catabolic process	28	373	1.23	1.23E-23
	(GO:0043603) Cellular amide metabolic process	34	773	1	2.74E-22
	(GO:0072594) Establishment of protein localization to organelle	28	433	1.17	4.94E-22
	(GO:1901566) Organonitrogen compound biosynthetic process	38	1346	0.81	5.86E-19
	(GO:0016071) mRNA metabolic process	29	678	0.99	3.07E-18
	(GO:0033365) Protein localization to organelle	29	743	0.95	3.31E-17
	(GO:0044271) Cellular nitrogen compound biosynthetic process	38	1522	0.75	3.4E-17
	(GO:0009057) Macromolecule catabolic process	32	1058	0.84	3.46E-16
	(GO:0006886) Intracellular protein transport	31	999	0.85	6.87E-16
	(GO:0044265) Cellular macromolecule catabolic process	30	917	0.87	6.87E-16
	(GO:0051649) Establishment of localization in cell	44	2375	0.62	7.49E-16
	(GO:0042254) Ribosome biogenesis	20	292	1.19	1.35E-15
	Reactome pathways				
	(HSA-156827) L13a-mediated translational silencing of Ceruloplasmin expression	29	108	1.79	1.3E-38
	(HSA-72706) GTP hydrolysis and joining of the 60S ribosomal subunit	29	109	1.78	1.3E-38
	(HSA-156902) Peptide chain elongation	27	87	1.85	7.96E-38
	(HSA-192823) Viral mRNA Translation	27	87	1.85	7.96E-38
	(HSA-2408557) Selenocysteine synthesis	27	91	1.83	1.61E-37
	(HSA-72764) Eukaryotic Translation Termination	27	91	1.83	1.61E-37
	(HSA-975956) Nonsense Mediated Decay (NMD) independent of the Exon Junction Complex (EJC)	27	93	1.82	1.87E-37
	(HSA-72689) Formation of a pool of free 40S subunits	27	99	1.79	7.35E-37
	(HSA-9633012) Response of EIF2AK4 (GCN2) to amino acid deficiency	27	99	1.79	7.35E-37
	(HSA-1799339) SRP-dependent cotranslational protein targeting to membrane	27	110	1.75	7.48E-36
	(HSA-168255) Influenza Infection	29	154	1.63	8.42E-36
	(HSA-975957) Nonsense Mediated Decay (NMD) enhanced by the Exon Junction Complex (EJC)	27	113	1.74	1.23E-35
	(HSA-376176) Signaling by ROBO receptors	31	213	1.52	1.79E-35
	(HSA-9010553) Regulation of expression of SLITs and ROBOs	29	167	1.6	5.16E-35
	(HSA-71291) Metabolism of amino acids and derivatives	34	365	1.33	2.83E-33
	(HSA-6791226) Major pathway of rRNA processing in the nucleolus and cytosol	27	180	1.53	6.59E-31
	(HSA-2262752) Cellular responses to stress	35	544	1.17	3.12E-29
	(HSA-422475) Axon guidance	35	547	1.16	3.59E-29
	(HSA-5663205) Infectious disease	39	826	1.03	2.54E-28
	(HSA-8953854) Metabolism of RNA	33	659	1.06	4.1E-24

Module ID (color)	(term ID) term description	observed gene count	background gene count	strength	false discovery rate
	GO Biological process				
WM2 (turquoise)	(GO:0002474) Antigen processing and presentation of peptide antigen via mhc class i	23	96	0.96	1.07E-11
	(GO:0042590) Antigen processing and presentation of exogenous peptide antigen via mhc class i	20	80	0.98	1.91E-10
	(GO:0002479) Antigen processing and presentation of exogenous peptide antigen via mhc class i, tap-dependent	19	75	0.99	5.23E-10
	(GO:0006521) Regulation of cellular amino acid metabolic process	17	64	1.01	3.44E-09
	(GO:0038061) NIK/NF-kappaB signaling	18	81	0.93	1.02E-08
	(GO:0033238) Regulation of cellular amine metabolic process	18	82	0.93	1.18E-08
	(GO:1902036) Regulation of hematopoietic stem cell differentiation	17	74	0.95	2.15E-08
	(GO:0010972) Negative regulation of g2/m transition of mitotic cell cycle	18	92	0.88	5.47E-08
	(GO:0031145) Anaphase-promoting complex-dependent catabolic process	17	83	0.9	8.92E-08
	(GO:0060071) Wnt signaling pathway, planar cell polarity pathway	18	96	0.86	9.46E-08
	(GO:0061418) Regulation of transcription from rna polymerase ii promoter in response to hypoxia	16	78	0.9	2.34E-07
	(GO:0006635) Fatty acid beta-oxidation	14	56	0.98	2.65E-07
	(GO:0009060) Aerobic respiration	16	79	0.89	2.65E-07
	(GO:0031146) SCF-dependent proteasomal ubiquitin-dependent protein catabolic process	17	93	0.85	3.14E-07
	(GO:0070498) interleukin-1-mediated signaling pathway	17	96	0.83	4.57E-07
	(GO:0006614) SRP-dependent cotranslational protein targeting to membrane	16	96	0.81	2.23E-06
	(GO:0010499) Proteasomal ubiquitin-independent protein catabolic process	8	22	1.15	3.87E-05
	(GO:0006090) Pyruvate metabolic process	12	69	0.83	5.87E-05
	(GO:0002181) Cytoplasmic translation	12	72	0.81	8.58E-05
	(GO:0051238) Sequestering of metal ion	6	11	1.32	1.50E-04
	Reactome pathways				
	(HSA-9010553) Regulation of expression of SLITs and ROBOs	32	167	0.87	3.51E-14
	(HSA-5678895) Defective CFTR causes cystic fibrosis	19	59	1.09	1.13E-11
	(HSA-5619084) ABC transporter disorders	20	75	1.01	4.44E-11
	(HSA-450408) AUF1 (hnRNP D0) binds and destabilizes mRNA	17	53	1.09	1.87E-10
	(HSA-5362768) Hh mutants are degraded by ERAD	17	54	1.08	2.12E-10
	(HSA-4641258) Degradation of DVL	17	55	1.07	2.43E-10
	(HSA-1236978) Cross-presentation of soluble exogenous antigens (endosomes)	16	48	1.11	3.73E-10
	(HSA-211733) Regulation of activated PAK-2p34 by proteasome mediated degradation	16	48	1.11	3.73E-10
	(HSA-382556) ABC-family proteins mediated transport	21	101	0.9	3.73E-10
	(HSA-5676590) NIK-->noncanonical NF-kB signaling	17	57	1.06	3.73E-10
	(HSA-5607761) Dectin-1 mediated noncanonical NF-kB signaling	17	58	1.05	3.77E-10
	(HSA-350562) Regulation of ornithine decarboxylase (ODC)	16	49	1.1	3.98E-10
	(HSA-162909) Host Interactions of HIV factors	23	129	0.84	4.73E-10
	(HSA-180534) Vpu mediated degradation of CD4	16	50	1.09	4.73E-10
	(HSA-349425) Autodegradation of the E3 ubiquitin ligase COP1	16	50	1.09	4.73E-10
	(HSA-69601) Ubiquitin Mediated Degradation of Phosphorylated Cdc25A	16	50	1.09	4.73E-10
	(HSA-72737) Cap-dependent Translation Initiation	22	116	0.86	4.73E-10
	(HSA-75815) Ubiquitin-dependent degradation of Cyclin D	16	50	1.09	4.73E-10
	(HSA-180585) Vif-mediated degradation of APOBEC3G	16	52	1.07	5.92E-10
	(HSA-8941858) Regulation of RUNX3 expression and activity	16	52	1.07	5.92E-10
